# The little-known genus *Dahliphora* Schmitz, 1923 of China (Diptera, Phoridae)

**DOI:** 10.3897/zookeys.714.15927

**Published:** 2017-11-07

**Authors:** Guang-Chun Liu

**Affiliations:** 1 Key Laboratory of Urban Integrated Pest Management and Ecological Security of Liaoning Province, College of Life Science and Bioengineering, Shenyang University, Shenyang 110044, China

**Keywords:** *Dahliphora chaetocauda*, identification key, morphological data, new record, new species, scuttle fly

## Abstract

The genus *Dahliphora* Schmitz, 1923 is recorded from China for the first time. Three *Dahliphora* species are reported, namely *D. sigmoides*, *D. zaitzevi*, and a new species here described, *D. chaetocauda* **sp. n.** Some new morphological data are reviewed and illustrated for the first time, and an identification key to species present in China is presented.

## Introduction

The small genus *Dahliphora* Schmitz, 1923 is one of the least well known genera in the family Phoridae (Diptera). All species of the genus are tiny and with less than 1 mm body length. At present only five species are known, namely *D. sigmoides* Schmitz, 1923 from the Bismarck Archipelago (Schmitz 1923, 1928) and Malaysia (Zuha et al. 2014); *D. crenaticornis* Borgmeier, 1961 and *D. dispar* Borgmeier, 1961 from Brazil and Dominica (Borgmeier 1961, 1969), and Mali (Mostovski, pers. comm. 2017); *D. antennalis* Borgmeier & Prado, 1975 from Ecuador (Borgmeier and do Prado 1975); and *D. zaitzevi* Michailovskaya, 2002 from Russia Far East (Michailovskaya 2002). The life cycles of most species are unknown, except that *D. sigmoides* was found on an animal carcass (Zuha et al. 2014). The genus is characterized by the presence of pseudo-arista or a long, thick, and unsegmented arista in male antenna, reduction of frons bristles and absence of isolated bristles and hair palisades of the mid and hind tibia. The supra-generic classification of *Dahliphora* was less involved. Schmitz (1923) classified the genus in subfamily Metopinae and considered that it is closely related with *Metopina*, based on the similarity of wing veins (Schmitz 1928, 1929). This arrangement was followed by Borgmeier (1961, 1969) and Borgmeier and do Prado (1975). A further morphological study will be needed to better understand its relationship. In present paper, the genus *Dahliphora* is reported from China for the first time, with three recorded species, one of them new to science and described here. Some new morphological data are firstly reviewed and illustrated, and an identification key to the males of the known species of the Oriental and Palaearctic regions is presented.

## Materials and methods

Since 2001, a series of specimen collection has been made by author and his team for studying Chinese phorid fauna and the project was funded by the National Nature Science Foundation of China. Specimens were collected into 80% ethanol using sweep nets and Malaise traps. The head, legs and wing were detached and made slides according to the method of Disney (1994). Line drawings were made using Leica M205C with a drawing appendage. Photos were made using microscope Leica M205A and Leica DM2500B with the help of a CCD 450 multi-focus imaging system. The terms used was followed Schmitz (1938) and modified by Disney (1994). The species recognition is mostly based on male specimens, as is typical for treatments of this genus. Males and females are dimorphic in many characters, and cannot be confidently associated in most situations. The type specimens are deposited in Natural History Museum of Shenyang University (**NMSU**), Shenyang, China.

## Results

### Key to Oriental and Palaearctic species (males only)

**Table:** 

1	Antenna postpedicel drawn out a long pseudo-arista, without arista (Fig. 7); frons with pre-ocellar bristles	**2**
–	Antenna postpedicel onion-form, with a long, thick and unsegmented arista (Fig. 4); frons without pre-ocellar bristles (Fig. 3); four subequal scutellar bristles; costa with 19–21dorsal cilia; thin veins very obscure (Figs 9–10)	***D. chaetocauda* sp. n.**
2	Notopleura with two bristles; hind metatarsus with four transverse hair combs (Fig. 8); costa with 16 dorsal cilia (Fig. 11); wing 0.55 mm long	***D. sigmoides* Schmitz**
–	Notopleura with three bristles; hind metatarsus with five transverse hair combs(Fig. 6); costa with 20 dorsal cilia (Fig. 12); wing 0.64 mm long	***D. zaitzevi* Michailovskaya**

### Taxonomy

#### Dahliphora chaetocauda sp. n.

Taxon classificationAnimaliaDipteraPhoridae

http://zoobank.org/CFFA065F-CD5D-471D-B1A4-E17415D527E8

Figs 1–6 , 9–10 , 13–14

##### Diagnosis.

Male. Frons without pre-ocellar bristles; postpedicel onion-form, with a long thick and unsegmented arista; notopleura with three bristles; scutellum with four subequal bristles; costa with 19–21 dorsal cilia; wing hyaline, thin veins very obscure. Female. Frons with two supra-antennal bristles.

##### Description.

**Figures 1–8.**
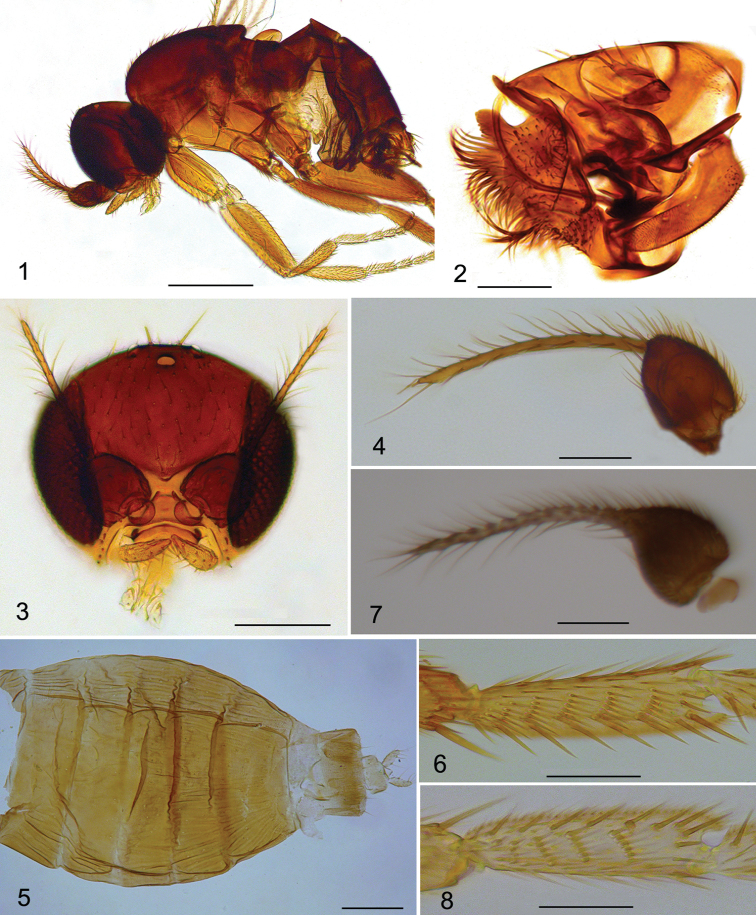
*Dahliphora* species. **1–6** *D. chaetocauda*. **1** body, male, lateral view **2** hypopygium, posterior view **3** head, male, frontal view **4** antenna, male **5** abdominal tergites, female, dorsal view **6** hind metatarsus, male, ventral view **7** *D. zaitzevi*, male, antenna **8** *D. sigmoides*, male, hind metatarsus, ventral view. Scale bars 0.2 mm (**1, 5**); scale bars 0.05 mm (**2–4**, **6–8**).

**Figures 9–12.**
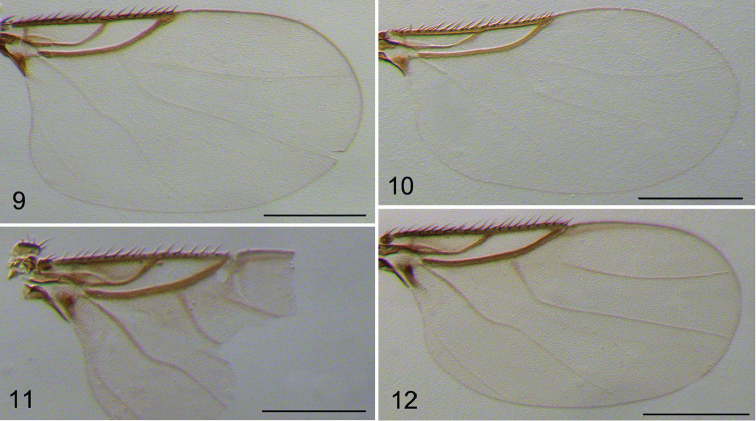
Wings. **9–10** *D. chaetocauda*. **9** male **10** female **11** *D. sigmoides*, male **12** *D. zaitzevi*, male. Scale bars 0.2 mm.

**Figures 13–16.**
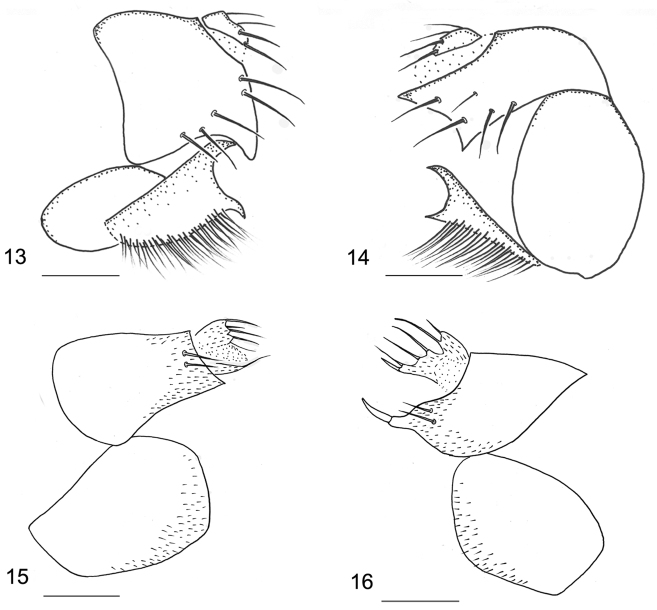
Hypopygia. **13–14** *D. chaetocauda*. **13** left view **14** right view **15–16** *D. zaitzevi* **15** left view **16** right view. Scale bars 0.05 mm.

*Male.* Body (Fig. 1) brown, 0.81–0.82 mm long. Frons brown, covered with dense microtrichia and about 70–80 hairs. Frons (Fig. 3) bristles reduced, only two ocellar bristles and two convergent postero-lateral bristles present. Postpedicel (Fig. 4) brown and onion-shaped, with a long, thick and unsegmented arista, bearing sparsely long hairs. Palpus yellow, 0.1 mm long and 0.02 mm wide, with four long and thin bristles on apex and some short hairs along lower margin. Thorax brown. Mesopleuron bare. Notopleura with three bristles. Scutellum with four subequal bristles. Legs yellow, only hind femur light brown. Front tarsus with postero-dorsal hair palisade on tarsomeres 1–4, tarsomere 5 longer than tarsomere 4. Mid and hind tibiae without dorsal longitudinal hair palisades. Hind metatarsus (Fig. 6) with five transverse hair combs. Wing (Fig. 9) 0.66–0.68 mm long. Membrane hyaline, thin veins very obscure and almost inconspicuous. Costal index 0.47–0.48. Costal ratio 1.08–1.10:1. Costa with 19–21 dorsal cilia and each of them approx. 0.03 mm long. Vein sc free. No hair at base of Rs. Vein M_2_ strongly curved near base, distal half nearly straight. Vein CuA_1_ slightly S-form. No axillary bristles. Halter brown. Abdominal tergites brown, wider than long, with sparse short hairs along rear margin. Venter yellow, with tiny sparse microtrichia. Hypopygium (Fig. 2) yellowish brown, asymmetrical. Epandrium with short hairs and five to six bristles. Hypandrium (Figs 13, 14) bifurcated. Left hypandrial lobe with a large and long-hairy process, which strongly excavated ventrally. Aedeagus complex drawn out in a long curved process. Anal tube short.

*Female.* Body (Fig. 5) 0.83–0.88 mm long. Similar to the male, but differs as follows: frons with a pair of supra-antennal bristles. Postpedicel rounded, with 3-segmented apical arista. Palpus 0.08 mm long, 0.02 mm wide. Wing (Fig. 10) 0.66–0.68mm long. Costal index 0.48. Costal ratio 0.94:1. Costa with 15–16 dorsal cilia and each of them about 0.03 mm long. No Dufour’s crop mechanism and abdominal glands discharge.

##### Etymology.

The species name refers to the character of left hypopygial lobe. To be treated as an adjective.

##### Material examined.

*Holotype*, ♂, China, Yunnan, Ruili (24°6′36.55″N, 97°19′12.53″E; 960 m), 04–Aug–2009, Jian-Feng Wang. *Paratypes*, 84 ♂ and 26 ♀, same data as holotype.

##### Remarks.

In the key to world species (Borgmeier and do Prado 1975), this new species runs to couplet 2 to *D. dispar* (described from Brazil and Dominica). It can be differentiated from the latter by the frons without pre-ocellar bristles, wing costa with 19–21 dorsal cilia, vein sc shorter and longer setation on hypopygial lobe.

#### Dahliphora sigmoides

Taxon classificationAnimaliaDipteraPhoridae

Schmitz, 1923

Figs 8 , 11

Dahliphora sigmoides Schmitz, 1923: 188.

##### Diagnosis.

Male. Body brown, 0.68 mm long. Frons with two pre-ocellar bristles, two ocellar bristles and two convergent postero-laterial bristles. Postpedicel brown and drawn out a long, apical pseudo-arista. Palpus yellow. Thorax brown. Notopleura with two bristles. Scutellum with four subequal bristles. Legs yellow. Hind metatarsus (Fig. 8) with four transverse hair combs, the basal hair comb has only three hairs in a row. Wing (Fig. 11) 0.55 mm long. Membrane nearly hyaline, thin veins whitish yellow. Costal index 0.49. Costal ratio 1:1. Costa with 16 dorsal cilia and each of them approx. 0.03 mm long. Vein sc free. No hair at base of Rs. Vein M_2_ strongly curved near base, distal half nearly straight. Vein CuA_1_ slightly S-form. Vein A_2_ almost inconspicuous. Abdominal tergites brown, wider than long, with sparse short hairs along the rear margin. Venter yellow, with tiny sparse microtrichia. Hypopygium yellowish brown, asymmetrical. Epandrium without bristles. Aedeagus complex drawn out a long, curved process. Anal tube short.

##### Material examined.

1 ♂, China, Guangxi, Shiwandashan (21°54′40.01″N, 107°54′51.18″E; 684 m), 18–Aug–2011, Jian-Feng Wang.

##### Remarks.

This species is similar to *D. zaitzevi* (described from Russian Far East, see below). It can be distinguished from the latter in having two bristles on notopleura, four transverse hair combs on hind metatarsus, 16 dorsal costal cilia and by smaller size.

#### Dahliphora zaitzevi

Taxon classificationAnimaliaDipteraPhoridae

Michailovskaya, 2002

Figs 7 , 12 , 15 , 16

Dahliphora zaitzevi Michailovskaya, 2002: 1.

##### Diagnosis.

Male. Body brown, 0.78 mm long. Frons brown, with two pre-ocellar bristles, two ocellar bristles and two convergent postero-laterial bristles. Postpedicel (Fig. 7) brown and drawn out a long, apical pseudo-arista. Thorax brown. Notopleura with three bristles. Scutellum with four subequal bristles. Legs yellow, only hind femur light brown. Hind metatarsus with five transverse hair combs. Wing (Fig. 12) 0.64 mm long. Membrane nearly hyaline, thin veins whitish yellow. Costal index 0.5. Costal ratio 1.0:1.1. Costa with 20 dorsal cilia and each of them approx. 0.03 mm long. Vein M_2_ strongly curved near base, distal half nearly straight. Vein CuA_1_ slightly S-form. Vein A_2_ almost inconspicuous. Halter brown. Abdominal tergites brown, wider than long, with sparse short hairs along the rear margin. Venter yellow, with tiny sparse microtrichia. Hypopygium (Figs 15, 16) yellowish brown, asymmetrical. Epandrium with two or three bristle-like hairs on each side. The tip of right epandrium with a very strong bristle. Aedeagus complex drawn out in a long, curved process.

##### Material examined.

1 ♂, China, Liaoning, Mt. Qianshan (40°59′44.58″N, 123°07′23.85″E; 590 m), 31–Aug–2013, Zhuo Zhang; 1 ♂, China, Jilin, Huicun (42°54′27.33″N, 130°50′25.19″E; 164 m), 3–Aug–2014, Jian-Feng Wang.

##### Remarks.

This species is similar to *D. sigmoides*, differing from the latter by three bristles on notopleura, five transverse hair combs on hind metatarsus, 21 dorsal costal cilia, and a larger size. *Dahliphora zaitzevi* is the only species of the genus which is distributed in the temperate area.

## Supplementary Material

XML Treatment for Dahliphora chaetocauda

XML Treatment for Dahliphora sigmoides

XML Treatment for Dahliphora zaitzevi
